# Rectangular Augmented Row–Column Designs Generated From Contractions

**DOI:** 10.1002/bimj.70182

**Published:** 2026-09-24

**Authors:** Hans‐Peter Piepho, Emlyn Williams

**Affiliations:** ^1^ Biostatistics Unit Institute of Crop Science University of Hohenheim Stuttgart Germany; ^2^ Statistical Support Network Australian National University Canberra Australian Capital Territory Australia

**Keywords:** 96‐well plate, auxiliary design, average efficiency factor, general balance, optimal design, microplate

## Abstract

Row–column designs play an important role in applications where two orthogonal sources of error need to be controlled for by blocking. Field or greenhouse experiments, in which experimental units are arranged as a rectangular array of experimental units, are a prominent example. In plant breeding, the amount of seed available for the treatments to be tested may be so limited that only one experimental unit per treatment can be accommodated. In such settings, augmented designs become an interesting option, where a small set of treatments, for which sufficient seed is available, is replicated across the rectangular layout so that row and column effects, as well as the error variance, can be estimated. Here, we consider the use of an auxiliary design, also known as a contraction, to generate an augmented row–column design. We make use of the fact that the efficiency factors of the contraction and the associated augmented design are closely interlinked. A major advantage of this approach is that an efficient contraction can be found by computer search at much higher computational speed than is required for direct search for an efficient augmented design. Two examples are used to illustrate the proposed method. Furthermore, we contrast the usage of our approach when the array dimensions are fixed, as is the case with microplates, to settings with more flexibility regarding the number of rows and columns of the layout, as in greenhouse and field trials.

## 1 Introduction

Field experiments laid out on a rectangular grid of plots often exhibit patterns of heterogeneity between experimental units that call for blocking along both rows and columns of the layout. Row–column designs are also relevant in other contexts where experimental units are arranged on a rectangular grid, such as greenhouse experiments or studies involving assays with microplates, such as 96‐well plates.

Most experiments laid out according to row–column designs are fully replicated. In cases where material for most of the treatments is limited, meaning that these treatments cannot be replicated, augmented designs (Federer 1956, 1961) may be used, in which only a small set of treatments, referred to here as checks, are replicated, whereas the remaining treatments, referred to here as test lines, are evaluated on only one experimental unit each. Such applications are common, for example, in early‐generation plant breeding trials where seed availability for the test lines is limited, precluding a fully replicated design. The original proposal for augmented designs only involved one‐way blocking. Extensions to row‐and‐column blocking were subsequently considered in several publications, including Federer and Raghavarao (1975), Lin and Poushinsky (1983), Federer and Crossa (2005), Piepho and Williams (2016), and Vo‐Thanh and Piepho (2020). With augmented row‐column designs, all information on the effects of row and column blocks, as well as on the residual error variance, stems from the replicated checks if the linear model used for analysis has fixed treatment effects. Hence, it is important to ensure connectivity of the design through the checks.

Recently, Bailey and Haines (2025) considered the construction of augmented designs in square arrays from smaller auxiliary designs, also known as contractions (Patterson and Williams 1976). A key idea used by the authors is a link between the average pairwise variance of treatment differences of the augmented design and its contraction. Also focusing on designs laid out on a square array of experimental units, Williams and Piepho (2025) compared cyclic designs with computer‐generated designs for the contraction, finding that the cyclic and computer‐generated augmented designs agree in some settings, whereas in other settings computer‐generated designs provide an edge in efficiency.

Designs laid out as square arrays have their role in practice (Boyhn et al. 2023), but in many applications the requirement of a square layout is too restrictive. For example, 96‐well microplates have a rectangular 12 × 8 layout, and this is by far the most frequently used layout for microplates. Other rectangular layouts are sometimes used for a larger number of wells, such as 24 × 16 for 384‐, 48 × 32 for 1536‐, and 72 × 48 for 3456‐well plates, respectively. Similarly, the layout of field experiments is seldom a square but often a rectangle. Hence, there is a need for methods of constructing rectangular augmented row–column designs providing flexibility in terms of the numbers of rows and columns, as well as the number of checks and test lines, and the proportion of plots allocated to checks. Here, we consider such methods based on the idea of using contractions as proposed in Bailey and Haines (2025). The main advantage of generating an augmented design from its contraction, as compared to direct computer‐aided design generation, is the speed of construction and in particular the facility to attain good average efficiency factors very quickly. This is because the contractions are typically quite small and so design generation packages using an iterative approach to optimize the average efficiency factor can stabilize on an optimal or near‐optimal design in a matter of seconds. On the other hand, augmented row–column designs are usually much larger and so the iterative process may not fully stabilize for many minutes, perhaps even hours. Of course, comparisons would depend on the design parameters and the hardware/software used. Our approach is based on the computer‐based generation of a near‐optimal contraction from which an augmented row–column design is obtained.

A practicality that is important to consider in generating a suitable design is the degree of flexibility regarding the number of rows and columns of the layout. When using microplates, the array dimensions are usually fixed and dictated by the laboratory equipment used. This means that the specification of the number of experimental units allocated to checks automatically fixes the number of test lines that can be tested on the same array. Hence, for a given proportion of experimental units to be allocated to checks, the only way to modify the number of test lines that can be evaluated is to increase the number of fixed arrays (microplates). By contrast, when there is more freedom to choose the array dimensions, as is usually the case in greenhouses and in field trials, the dimensions can be tailored to simultaneously accommodate the targeted percentage of experimental units devoted to checks, as well as the number of test lines that can be evaluated.

The remainder of this paper is structured as follows. In the next section, we state our main result, along with the proof, and provide two illustrative examples. In the third section, we consider special cases in which a direct link between the efficiency factors of an augmented design and those of its contraction can be established. The paper ends with a discussion, which provides several examples for both fixed and more flexible arrays and provides hints for the choice of design dimensions.

## 2 The Main Result

Our aim is to construct an efficient augmented design in a rectangle with v rows and s columns (v≥s). In each column of the design there will be k checks and (v−k) test lines, that is, (v−k)s test lines overall. The number of different checks will equal k, and each column of the augmented design will comprise one plot for each of the k checks. Each row will have $r_{h}\;\left(h=1\text{…}v\right)$ checks and $s-r_{h}$ test lines. Where the parameters v, s, and k allow, we aim for equal $r_{h}$ for each row of the augmented design; otherwise they will differ by no more than one.

The construction starts with a contraction for v pseudo‐treatments with the hth pseudo‐treatment replicated $r_{h}$ times in a k×s array, and we define $\;r^{\prime }=\left(r_{1}\text{…}r_{v}\right)$. We assume that the contraction is a binary design and look for an optimal or near‐optimal row–column design. Note that here we use the term “pseudo‐treatments” for the components of the contraction, rather than the more common term “treatments,” and that these v pseudo‐treatments serve as an auxiliary device to allocate checks to the plots of the augmented design; they are not identical to the $v^{*}$ treatments of the augmented design obtained from the contraction. For the augmented design, we refer to the treatments as “test lines” and “checks.”

From the contraction, an augmented row–column design can be constructed in a v×s array as follows:

1. Label the test lines from 1 through (v−k)s
2. Label the checks from (v−k)s+1 through $v^{*}=\left(v-k\right)\;s+k$
3. If pseudo‐treatment l (l=1…v) is in position (i,j) of the contraction (i=1…k,j=1…s), then place check (v−k)s+i in position (l,j) of the augmented design
4. Fill the remaining (v−k)s empty positions of the augmented design with the test lines

For the augmented design, we assume the model

$$y=X\tau +Z_{R}\rho +Z_{C}\gamma +\varepsilon$$

where X, $Z_{R},$ and $Z_{C}$ are $n\times v^{*},$ n×v, and n×s design matrices for the effects of treatments, rows, and columns, respectively, with n=v×s. The information matrix is given by

$$A=u^{\delta }\;-\frac{1}{s}M_{R}M_{R}^{\prime }-\frac{1}{v}M_{C}M_{C}^{\prime }+uu^{\prime }$$

(John and Williams, 1995, eq. 5.3), where u is a vector of treatment replications, in order, with checks at the end, that is, $\;u^{\prime }=\left(1_{(v-k)s}^{\prime },s1_{k}^{\prime }\right),\;M_{R}=X^{\prime }Z_{R},$ $M_{C}=X^{\prime }Z_{C}$, and $u^{\delta }=diag\left(u\right)$. We are interested in the canonical efficiency factors (*cefs*) of $A^{*}=u^{-1/2}{Au}^{-1/2}$ (John and Williams, 1995, eq. 5.5) which we can write as

$$\;A^{*}=I_{v^{*}}-u^{-\delta /2}X^{\prime }\left[Z_{R}Z_{C}\right]{\left(\begin{array}{cc} sI_{v} & 0\\ 0 & vI_{s} \end{array}\right)}^{-1}\left[\begin{array}{c} Z_{R}^{\prime }\\ Z_{C}^{\prime } \end{array}\right]Xu^{-\delta /2}+\frac{1}{v^{*}}J_{v^{*},v^{*}}$$

where $I_{v^{*}}$ is the identity matrix of size $v^{*}$ and $J_{v^{*},v^{*}}$ is the $v^{*}\times v^{*}$ matrix of ones. The $v^{*}-1$ non‐trivial eigenvalues of the central term will be the same as those of

(1) $$D^{*}={\left(\begin{array}{cc} sI_{v} & 0\\ 0 & vI_{s} \end{array}\right)}^{-1/2}\left[\begin{array}{c} Z_{R}^{\prime }\\ Z_{C}^{\prime } \end{array}\right]Xu^{-\delta }X^{\prime }\left[Z_{R}Z_{C}\right]{\left(\begin{array}{cc} sI_{v} & 0\\ 0 & vI_{s} \end{array}\right)}^{-1/2}$$

along with $v^{*}-\left(v+s\right)-1$ zero eigenvalues.

This expression can be manipulated with the addition/subtraction of terms related to the trivial eigenvectors, for example, those of the form $\left(a1_{v}^{\prime },b1_{s}^{\prime }\right)$ for some (a, b); these correspond to trivial eigenvalues and so, such changes do not affect the *cefs* of the augmented row–column design. Hence, we can derive the form

(2) $$B^{*}={\left(\begin{array}{cc} sI_{v} & 0\\ 0 & vI_{s} \end{array}\right)}^{-1/2}\;\left[\begin{array}{cc} r^{\delta }-\frac{1}{s}W & N_{C}-\frac{1}{s}r^{\delta }J_{v,s}\\ N_{C}^{\prime }-\frac{1}{s}J_{s,v}r^{\delta } & kI_{s} \end{array}\right]{\left(\begin{array}{cc} sI_{v} & 0\\ 0 & vI_{s} \end{array}\right)}^{-1/2}$$

where $N_{C}$ is the v×s column incidence matrix for the contraction and $W=N_{R}N_{R}^{\prime }$ is its v×v row concurrence matrix. In particular,

$$\;B^{*}=I_{\left(v+s\right)}\;-D^{*}+\left[\begin{array}{cc} 0 & \frac{1}{\sqrt{vs}}J_{v,s}\\ \frac{1}{\sqrt{vs}}J_{s,v} & \frac{k}{vs}J_{s,s} \end{array}\right]$$

The (v−1)+(s−1) non‐trivial eigenvalues of $B^{*}$ are eigenvalues (*cefs*) of the scaled information matrix of the augmented design, namely $A^{*}$.

These *cefs* can then be combined with $v^{*}-\left(v-1\right)-\left(s-1\right)-1$ unit eigenvalues of $A^{*}$ in order to calculate the average efficiency factor of the augmented design, namely $E_{\mathrm{aug}}$. Hence, considering that $E_{\mathrm{aug}}$ is the harmonic mean of the *cefs*, we are therefore interested in the trace of the inverse of $B^{*}$. We now make use of a block matrix identity (Gubner 2024) to show that the diagonal blocks of ${B^{*}}^{-1}$ can be written as

(3) $$\frac{v}{k}\;{\left[r^{\delta }-\frac{1}{s}W-\frac{1}{k}\left(N_{C}-\frac{1}{s}r^{\delta }J_{v,s}\right)\left(N_{C}^{\prime }-\frac{1}{s}J_{s,v}r^{\delta }\right)\right]}^{-1}$$

and

(4) $$\frac{v}{k}\;{\left[I_{s}-\left(N_{C}^{\prime }-\frac{1}{s}J_{s,v}r^{\delta }\right){\left(r^{\delta }-W+J_{v,v}\right)}^{-1}\left(N_{C}-\frac{1}{s}r^{\delta }J_{v,s}\right)\right]}^{-1}$$

Note that an extra matrix $J_{v,v}$ has been added into the middle term in Equation (4) to make it non‐singular; this does not affect the relevant eigenvalues. After multiplying out and ignoring unimportant terms in $J_{v,v},$ we can recognize the expression within the square brackets of Equation (3) as the row–column information matrix of the contraction (John and Williams 1995, eq. 5.3). Let $C_{v}$ be the harmonic mean of the non‐trivial eigenvalues of the expression in square brackets of Equation (3) and $\bar{r}$ be the arithmetic average of the $r_{h}\;\left(h=1\text{…}v\right)$ pseudo‐treatment replication numbers. Then we define $\;{\bar{C}}_{v}=C_{v}/\bar{r}$. Note that for equal replication numbers, ${\bar{C}}_{v}$ becomes the average efficiency factor of the row–column contraction. In a similar manner, we define ${\bar{C}}_{s}$ as the harmonic mean of the non‐trivial eigenvalues of the expression in square brackets of (4). Expression (4) is a little more complicated but still involves components associated with the contraction and in particular there is a type of interaction between its row and column incidence matrices. With these definitions we can then establish a formula for the average efficiency factor of the augmented row–column design, that is,

(5) $$\;E_{\mathrm{aug}}=\frac{\left(v^{*}-1\right)}{\left\{v^{*}-\left(v+s\right)+1+\frac{v}{k}\left(\frac{v-1}{{\bar{C}}_{v}}+\frac{s-1}{{\bar{C}}_{s}}\right)\right\}}\;$$

We now consider two examples of how contractions are used to produce efficient augmented designs. The examples correspond to designs that can be used for 96‐ and 384‐well plates. All designs considered in this paper were produced using the design generation package CycDesigN (VSNi 2025), which constructs optimal or near‐optimal designs as measured by the average efficiency factor. When constructing row–column designs, the package checks that the parameters are such that a connected design is possible. For example, we want to ensure there are non‐negative degrees of freedom in the fixed‐effects row–column analysis of variance table of the contraction, that is,

(6) $$ks-1-\left(k-1\right)-\left(s-1\right)-\left(v-1\right)\geq 0$$

**Example 1**

For a contraction with v=12,s=8,k=3 and equal pseudo‐treatment replication, that is, $r_{h}=2$ for all h and hence $\bar{r}=2$. The package gives the contraction

**Table:** 

	Column							
	3	7	9	1	10	8	2	6
**Row**	12	10	4	3	11	9	5	2
	7	5	6	4	1	11	8	12

with ${\bar{C}}_{v}=0.5739$ and ${\bar{C}}_{s}=0.4828$. The resulting augmented row–column design is the following 12×8 array:

**Table:** 

	Column							
	1	10	19	73	75	46	55	64
	2	11	20	28	37	47	73	74
	73	12	21	74	38	48	56	65
	3	13	74	75	39	49	57	66
	4	75	22	29	40	50	74	67
**Row**	5	14	75	30	41	51	58	73
	75	73	23	31	42	52	59	68
	6	15	24	32	43	73	75	69
	7	16	73	33	44	74	60	70
	8	74	25	34	73	53	61	71
	9	17	26	35	74	75	62	72
	74	18	27	36	45	54	63	75

This has three checks (numbered 73, 74, and 75) in each column and two checks in each row of the array and has an average efficiency factor of $E_{\mathrm{aug}}=0.3881$.

For comparison, we used the augmented design option in the package to generate the full array directly (Supporting Information A), giving $E_{\mathrm{aug}}=0.3860$. This shows that the design generated from the contraction is very competitive, providing an edge in efficiency.

**Example 2**

For a contraction with v=24,s=16,k=5 and pseudo‐treatment replications of three or four with $\bar{r}=3.3,$ the package gives the contraction

**Table:** 

	Column															
	13	20	7	8	19	15	9	23	11	2	3	18	10	24	16	1
	6	15	9	10	12	1	14	18	23	17	5	3	21	16	22	8
**Row**	21	4	19	22	15	5	11	2	17	9	8	14	24	6	13	18
	20	9	5	23	21	10	13	6	7	24	17	15	4	8	18	12
	1	22	6	19	16	2	10	4	21	12	20	7	3	14	17	11

with ${\bar{C}}_{v}=0.7749$ and ${\bar{C}}_{s}=0.7332$. The resulting augmented rectangular design is the following 24×16 array:

**Table:** 

	Column															
	309	20	39	58	77	306	115	134	153	172	191	210	229	248	267	305
	1	21	40	59	78	309	116	307	154	305	192	211	230	249	268	286
	2	22	41	60	79	96	117	135	155	173	305	306	309	250	269	287
	3	307	42	61	80	97	118	309	156	174	193	212	308	251	270	288
	4	23	308	62	81	307	119	136	157	175	306	213	231	252	271	289
	306	24	309	63	82	98	120	308	158	176	194	214	232	307	272	290
	5	25	305	64	83	99	121	137	308	177	195	309	233	253	273	291
	6	26	43	305	84	100	122	138	159	178	307	215	234	308	274	306
	7	308	306	65	85	101	305	139	160	307	196	216	235	254	275	292
	8	27	44	306	86	308	309	140	161	179	197	217	305	255	276	293
	9	28	45	66	87	102	307	141	305	180	198	218	236	256	277	309
**Row**	10	29	46	67	306	103	123	142	162	309	199	219	237	257	278	308
	305	30	47	68	88	104	308	143	163	181	200	220	238	258	307	294
	11	31	48	69	89	105	306	144	164	182	201	307	239	309	279	295
	12	306	49	70	307	305	124	145	165	183	202	308	240	259	280	296
	13	32	50	71	309	106	125	146	166	184	203	221	241	306	305	297
	14	33	51	72	90	107	126	147	307	306	308	222	242	260	309	298
	15	34	52	73	91	108	127	306	167	185	204	305	243	261	308	307
	16	35	307	309	305	109	128	148	168	186	205	223	244	262	281	299
	308	305	53	74	92	110	129	149	169	187	309	224	245	263	282	300
	307	36	54	75	308	111	130	150	309	188	206	225	306	264	283	301
	17	309	55	307	93	112	131	151	170	189	207	226	246	265	306	302
	18	37	56	308	94	113	132	305	306	190	208	227	247	266	284	303
	19	38	57	76	95	114	133	152	171	308	209	228	307	305	285	304

This has five checks (numbered 305, 306, 307, 308, and 309) in each column and either three or four checks in each row of the array and has an average efficiency factor of $E_{\mathrm{aug}}=0.6031$.

Using the augmented design option in the package to generate the full array directly gives $E_{\mathrm{aug}}=0.6026$; this is after an extended period of searching (Supporting Information A). This demonstrates the advantage of using contractions to generate augmented designs, especially for larger situations.

## 3 Special Cases

The expression (5) shows the general connection between a contraction and the resulting augmented design. Here we discuss some special cases which allow a simplification of expression (5) and hence accentuate this link:

1. The pseudo‐treatments in the contraction are equally replicated, say $\bar{r}$ times, that is, $\bar{r}=ks/v$. This corresponds to the rows of the augmented design all having $\bar{r}$ checks, which is a desirable feature. In such a case, ${\bar{C}}_{v}$ will equal the average efficiency factor of the row–column contraction $\left(E_{\mathrm{con}}\right)$. In Table 1, we have listed the parameters of 21 contractions for v>s, where pseudo‐treatments are all replicated $\bar{r}$ times. These contractions generate augmented designs where:
  (i) The number of checks is k = 3, 4, or 5, and there are k check plots per column of the augmented rectangular array.
  (ii) The percentage of checks in the v×s array is between 15 and 25%; this is in fact the ratio k/v as a percentage.
2. Table 1 lists values for $E_{\mathrm{con}}$, ${\bar{C}}_{s}$, and the average efficiency factor for the dual design of the contraction $\left(E_{\mathrm{dual}}\right)$ when it is considered just as a column design. In 13 cases, $\;{\bar{C}}_{s}=E_{\mathrm{dual}}$ since $WN_{C}={\bar{r}}^{2}J_{v,s}$. Thus, the expression inside the square brackets of Equation (4) collapses to the equivalent of that for the dual of the column design for the contraction. This is related to the property of general balance (Nelder 1965), that is, the row and column concurrence matrices of the contraction commute (Williams and Bailey 2026). The final column in Table 1 lists values for $E_{\mathrm{aug}}$, as can be calculated from Equation (5). A list of the Table 1 contractions is given in Supporting Information B.

**TABLE 1:** Properties of contractions with equal replication of pseudo‐treatments and average efficiency factors for the resulting augmented designs.

k	v	s	$\bar{r}$	$E_{\mathrm{con}}$	${\bar{C}}_{s}$	$E_{\mathrm{dual}}$	$E_{\mathrm{aug}}$
3	12	8	2	0.5739	0.4828	0.4828	0.388112
3	15	10	2	0.5359	0.4424	0.4467	0.368217
3	18	12	2	0.5135	0.4176	0.4205	0.356396
4	16	8	2	0.6618	0.5385	0.5385	0.450683
4	16	12	3	0.7547	0.7097	0.7097	0.560000
4	18	9	2	0.6479	0.5111	0.5111	0.441030
4	20	10	2	0.6423	0.5000	0.5000	0.437095
4	20	15	3	0.7339	0.6825	0.6825	0.549752
4	22	11	2	0.6338	0.4851	0.4851	0.431698
4	24	12	2	0.6310	0.4793	0.4793	0.429763
4	24	18	3	0.7203	0.6652	0.6652	0.543467
4	26	13	2	0.6232	0.4688	0.4688	0.425538
5	20	8	2	0.6976	0.5453	0.5453	0.480081
5	20	12	3	0.7881	0.7201	0.7213	0.590627
5	20	16	4	0.8244	0.7993	0.8000	0.646791
5	25	10	2	0.6966	0.5294	0.5294	0.476348
5	25	15	3	0.7780	0.6992	0.7000	0.584213
5	25	20	4	0.8096	0.7801	0.7808	0.640252
5	30	12	2	0.6867	0.5038	0.5038	0.468846
5	30	18	3	0.7669	0.6805	0.6814	0.578506
5	30	24	4	0.7988	0.7661	0.7665	0.635813

We note that when features 1. and 2. hold, the objectives of optimizing $E_{\mathrm{con}}$ and $E_{\mathrm{aug}}$ are closely aligned. For example, when generating a row–column design, one may first maximize the average efficiency factor for just the columns and then with columns fixed, try to optimize the whole design. This strategy is particularly valuable for larger designs. With this approach, because the non‐unit *cefs* of the dual column design are the same as those of the column design (John and Williams 1995), using $E_{\mathrm{con}}$ as a criterion for optimization directly relates to the optimization of $E_{\mathrm{aug}}$. Furthermore, for designs with general balance, the *cefs* for the column design also comprise those for the row–column design. In general, though, using a package to search for near‐optimal contractions will result in near‐optimal augmented designs.

## 4 Discussion

This paper has proposed a method to obtain an augmented row–column design from a contraction, where the contraction is a computer‐generated near‐optimal design for pseudo‐treatments corresponding to the rows of the final design. It generalizes a method previously proposed by Bailey and Haines (2025) for square arrays to rectangular arrays. Their method is covered as a special case by our general result.

The extension to rectangular arrays greatly increases the options for the layout of the augmented design. However, there are some restrictions. For example, each column must have each check appearing on exactly one of the plots. This dictates that the proportion of plots allocated to checks must equal k/v, meaning that for a given number of rows (v) the choice of the number of checks determines the proportion of plots devoted to checks. If one wants to set the proportion of plots allocated to checks to a specific value, then the number of rows needs to be chosen accordingly. These choices need to be made appreciating that both k and v must be integers, which limits the possible values their ratio, and hence the values the proportion of plots devoted to checks can take.

If the number of rows and columns available for a layout is fixed, as is the case with microplates, options for design choice are more limited than in applications where there is some flexibility as to these dimensions, for example, in greenhouse trials and field experiments. With microplates of fixed dimension, there is only one choice, that is, the number of checks (k). This choice, taken together with the given number of rows and columns, determines the number of experimental units available for unreplicated test lines.

Consider a 96‐well plate where the layout is fixed to a 12 × 8 array. With v=12 and s=8 fixed, for k=2,3,4,5,6, the proportion of wells allocated to checks is $16.\bar{6}\%$, 25%, $33.\bar{3}\%$, $41.\bar{6}\%,$ and 50%, respectively, leaving 80, 72, 64, 56, and 48 plots for unreplicated test lines. For example, assume that there are three checks, meaning that 25% of wells are to be allocated to checks. The design will accommodate (v−k)s=(12−3)×8=72 test lines. Conversely, assume that we do not want to allocate more than 20% of the wells to checks. This can be accommodated by using only two checks, meaning that $k/v=2/12=16.\bar{6}\%$ of wells are devoted to checks and that (v−k)s=(12−2)×8=80 wells are available for test lines.

Next, assume we use a 24 × 16 = 384‐well plate and want to devote about 15% of the wells to checks. Hence, we might choose k=4, meaning that $k/v=4/24=16.\bar{6}\%$ of the wells will be devoted to four checks. This leaves space for (v−k)s=(24−4)×16=320 test lines. Alternatively, if we are willing to allocate more space to checks and use k=5 checks, we have k/v=5/24≈20.83%, and (v−k)s=(24−5)×16=304 test lines can be accommodated.

By comparison, there is relatively large flexibility regarding the number of test lines the design can accommodate if the number of rows and columns can be chosen more freely. The number of test lines needs to equal the product (v−k)s, so for given choice of v and k one can adjust the number of columns s such that the desired number of plots for test lines is obtained.

These restrictions suggest the following approach for choosing the dimensions of the trials, given by (v,s,k):

(i) Determine the number k of checks to be included in the trial.
(ii) Determine the approximate proportion of plots to be allocated to checks and find the value of v yielding the ratio k/v closest to the desired proportion.
(iii) Determine the number of test lines to be tested, making sure it is of the form (v−k)s. This determines the number of columns s of the augmented design.
(iv) For the choices of (v, s, k) found in (i) to (iii) generate an augmented design as described in Section 2.

To illustrate the approach, assume a breeder wants to use four checks and devote 20% of the trial to checks. Hence, with k = 4, the trial needs to have v = 20 rows such that k/v = 20% matches the desired proportion of plots devoted to checks. Further assume that 173 test lines are to be tested. The nearest multiple of v−k=20−4=16 is 11; hence, the trial needs to have 11 columns, providing space for (v−k)s=176 test lines. Thus, the breeder may add three further test lines to complete the design.

As a second example, assume that the number of columns or rows must be a multiple of six in order to accommodate spray lanes in the field layout, imposing a restriction on the design. Further assume that 654 test lines are to be evaluated and that the breeder would like to use five checks, devoting up to 10% of plots to these checks. Thus, the layout may be designed to have v=54 rows, meaning that 5/54 ≈ 9.3% of the plots are allocated to checks. Ideally, the number of test lines should then be a multiple of (v−k)=54−5=49. This suggests that to accommodate 654 test lines, the number of columns should be upward from 654/49 ≈ 13.3. Hence, we would need to set s=14, allowing space for 49 × 14 = 686 test lines. Thus, 32 test lines would need to be added to fill the layout. If this is decided to be too many, we may consider a layout with v=48 rows, such that 5/48 ≈ 10.4% of the plots are devoted to checks and the number of test lines should be a multiple of (v−k)=49−5=44. Observing that 654/44 ≈ 14.9, we may choose s=15, allowing space for 44 × 15 = 660 test lines. With these choices, only six additional test lines need to be added to fill the layout.

If the requirements for a planned augmented design do not allow finding a suitable specification (v, s, k), one can always resort to direct generation of an augmented row–column design, at the cost of more computing time (Piepho and Williams 2016; Vo‐Thanh and Piepho 2020). In future work, it may be explored if the idea to generate a design from a contraction can be extended to provide even more options, such as dropping the requirement to have each check present on one plot per column of the augmented row–column design, and also dropping the requirement to have the same number of replicates for each check.

The actual shape of the area covered by an array depends both on the number of rows and columns, as well as the shape of an experimental unit. The wells on microplates typically occupy a square area. By contrast, in field trials, plots are typically long and thin, in which case the overall arrangement of the plots in rows and columns can occupy a very non‐square area when the numbers of rows and columns are equal (or nearly equal), or equally an experiment might occupy a square area but with very different numbers of rows and columns. These properties need to be taken into account when selecting an area for field or greenhouse trials.

## Funding

The authors have nothing to report.

## Conflicts of Interest

The authors declare no conflicts of interest.

## Supporting information




**Supporting File 1:** bimj70182‐sup‐0001‐SuppMat A.xlsx.

**Supporting File 2:** bimj70182‐sup‐0002‐SuppMat B.docx.

**Supporting File 3:** bimj70182‐sup‐0003‐Data.zip.

## Data Availability

The data that support the findings of this study are available in the Supporting Information of this article.
