# Supplementary material for: Rectangular Augmented Row–Column Designs Generated From Contractions
Source: Biom J. 2026 Sep 24;68(5):e70182. doi: 10.1002/bimj.70182 (PMC13613013; doi:10.1002/bimj.70182)
Supplement: Supplementary file 1 [file BIMJ-68-e70182-s002.docx]

**Supplementary Material B – list of contraction designs in Table 1**

```
v=12,k=3,s=8


       column   1   2   3   4   5   6   7   8
          row
            1   3   7   9   1  10   8   2   6
            2  12  10   4   3  11   9   5   2
            3   7   5   6   4   1  11   8  12


v=15,k=3,s=10


       column   1   2   3   4   5   6   7   8   9  10
          row
            1   8   3  12   4   2   9  15   7  14  10
            2   4  11   7   6   3   5   9  13  10   1
            3   5  14   8  15   1   2  13  11   6  12


v=18,k=3,s=12


       column   1   2   3   4   5   6   7   8   9  10  11  12
          row
            1  14  12  10   7  15   4  11  17  18  16   5   6
            2   6  15   3   4   1   8   9  10  16  11  13   2
            3   8  13  12   1  14  18   7   9   3   5   2  17


v=16,k=4,s=8


       column   1   2   3   4   5   6   7   8
          row
            1   7  11  16   9   2   5   3  12
            2  10  13  12   8   1  15   9   4
            3   1  16   6   7  13   4  15  14
            4   5   8  10  14   3  11   6   2


v=16,k=4,s=12


       column   1   2   3   4   5   6   7   8   9  10  11  12
          row
            1  10   7  15   5  11   6   8   1  12  16   2   3
            2   3   4  14  10   9   8  16   6   2   1   7  13
            3   4  12   1   7   8  15  14   9   3  11  13   5
            4  11  16  10   9   2   4   5  12  14  13  15   6


v=18,k=4,s=9


       column   1   2   3   4   5   6   7   8   9
          row
            1   9  10   8  14   6   3   7   5   2
            2  14  17  10  15   1   9  18  11   7
            3  13   2  15  12  11   4   6   3  16
            4  16  13   4   5   8  18  12  17   1


v=20,k=4,s=10


       column   1   2   3   4   5   6   7   8   9  10
          row
            1   5   6  12  19  17   9  14  16  10   3
            2  15  18   8   7  11   6   5  20   1   2
            3  12   4  20   9  18  13   2  19  17   1
            4   4   3  10  15  14   8  13  11   7  16


v=20,k=4,s=15


       column   1   2   3   4   5   6   7   8   9  10  11  12  13  14  15
          row
            1  10   9   8  16   4   1   7  11   3  15  18  19   2  12  13
            2   8   6   5  17   2  19  13  20  15  12   1  10  16  18  14
            3  14   2   9  18  20  17  15   8   6   4   3   7   5  11  19
            4   4  13  12   7   1   9  20  16  10  17  14   5   3   6  11


v=22,k=4,s=11


       column   1   2   3   4   5   6   7   8   9  10  11
          row
            1  16  18   3  10   2  11  15  17  13  20   6
            2  20  19  22   9   1   3  10   7  12   8  13
            3   5   1  14  16  22   4   8  11  18  21   7
            4  19  15   6  14  17  12   4   9  21   2   5


v=24,k=4,s=12


       column   1   2   3   4   5   6   7   8   9  10  11  12
          row
            1   5  10  16   8  24  22   1  21  12  23   3  11
            2  23   2   4  24  13  17  11  14   9   6  22  15
            3  17   3   1  15  21  19  20   4   2  12   7  18
            4  18  14   9  19  20  16   6   5   8   7  13  10


v=24,k=4,s=18


       column  1  2  3  4  5  6  7  8  9 10 11 12 13 14 15 16  17  18
          row
            1  8 11 21 23 13  7 16 12  2 17 20  3 10  5 19  9  22   6
            2  4 18 23 12 14 21  7 10 20  9 13  8 16 19 15 22  24   1
            3  2  8 14 24 11 18  6  5 10  4 17  1  3 13 23 15   7   9
            4 19 12  3 17 16  5  4 15 14 21 18 22 24  1  6 11   2  20


v=26,k=4,s=13


       column   1   2   3   4   5   6   7   8   9  10  11  12  13
          row
            1   7  20   5   2  15   8   1  21  24  13  26  10  12
            2  21  14  12  24   3  15  25  16   6  18  23  17   4
            3  22  23  11  25   9  16  13  19  10  14   5   3   7
            4  18   2   6  19  20  11   4  26  22   8  17   1   9


v=20,k=5,s=8


       column   1   2   3   4   5   6   7   8
          row
            1  16  17  12   2   3   4  15  19
            2   9  20  10  19  15   8  18   5
            3   5  18   2   7   6   3   1  13
            4  17  13   8  11  10   7  16  14
            5   6  12   1   9  14  20  11   4


v=20,k=5,s=12


       column   1   2   3   4   5   6   7   8   9  10  11  12
          row
            1  10   6  19   9   8  20   3   4   7   1  13  17
            2  17  10   9   4  12   8  15   3   2  19  18  11
            3  15  16   5  12   7  11  19  18   4  14  20   6
            4   5   2  20  16   3   1  13   6  11  10  12  14
            5   7   8   2   1  14  15  16   5  13  18  17   9


v=20,k=5,s=16


       column  1  2  3  4  5  6  7  8  9 10 11 12 13 14 15 16
          row
            1  9 12 13 11  6  3 20  5 18  4 10  7 17  2  8 19
            2  7  4 18 10 20 16 12 19  3 11  6 15  8 14  1 13
            3  2 16 15 19  4 20  5  3  1  7 18 17  9 10 13 14
            4  3  2 11  9  1 17 13 15 12 14  5 10  4  8 16  6
            5  6 19  2  1 15 11  9  8 14  5 16 12 18 20  7 17


v=25,k=5,s=10


       column   1   2   3   4   5   6   7   8   9  10
          row
            1  10  14  23   7  17  12  20   8   4  25
            2  18  23  15   6  13  24  22   9  10   5
            3  19  16  21  12  11  13  15  20   3   4
            4   7   9  25  22  14   1   2  19  24  11
            5  21   3   1  16  18   8  17   5   2   6


v=25,k=5,s=15


       column   1   2   3   4   5   6   7   8   9  10  11  12  13  14  15
          row
            1  21   2  13  20  15  10  24  14  19  11   8  25   3   1   9
            2   8  23  15  18   7  11  13  22  25   2  17   3  10  16   6
            3   2   1  22  10  14  12   4  19   7   6  24   5   9  18  15
            4  22  25  12   5  23  17  11  16  18  20  14  24   4   9  21
            5   4  12   5   8  20  19   7   3  21  16   1   6  23  13  17


v=25,k=5,s=20


       column  1  2  3  4  5  6  7  8  9 10 11 12 13 14 15 16 17 18 19 20
          row
            1 10 25  9 23  6  8  7  3 18 15  5 20 12  4 17  1  2 22 16 21
            2  2  6 16 11 12  5 15 17  3 10 24 14  4 18 22  8  7 13  1 19
            3 22 13  3 17  9 11 25 24 23 19  2 12 21 16 14 20  6 15  5  8
            4 11 10  7  4 19 25 24 21  8  5  9  3 13 20 18  6 23  1 14 22
            5 20 16 11  1 17 12  4 10 13 23 18 15  2 19 25 24 14  9 21  7


v=30,k=5,s=12


       column   1   2   3   4   5   6   7   8   9  10  11  12
          row
            1  30  23  15  16   4   7  25   1  12  17  28  26
            2  14  11   5   2  22  29  13  23  18   6   7  16
            3   3  21  28  25  18  19  27  14  26  22  20  24
            4  12  19  11  10   1   3  15   9   8  27   2   6
            5   5  17  24  21  10   4  29  20  13  30   8   9


v=30,k=5,s=18


       column  1  2  3  4  5  6  7  8  9 10 11 12 13 14 15 16 17 18
          row
            1 28 13  7 23 21  4 29 16 17 24 22 25  8 30  9 12  3 18
            2 10 15 20 12 19  7 14  3 27  1 21 29 22 26 30  9  4  2
            3  7 20  6 13  2 23 24 14 30 15 11 16 10  1  5 17 22  4
            4 19  3 25  5 24 27 12 28  2  8  1 10 26 18 16 20  6 11
            5 13 18 21 26 23  8  6 27 25  9 28 15 17 14 19 11  5 29


v=30,k=5,s=24


          row   1   2   3   4   5
       column
            1   7  22  28  20   5
            2  26  24  30  14  15
            3  20  15   4  16   2
            4   3   7  29  18  30
            5  15  13   5   6  11
            6  27  18   6  22  21
            7  18  23   1  15  12
            8  13  19  26  12   7
            9  11   1  25  10  26
           10  10   2  18  28  17
           11  16   3  11   9  28
           12  29   4  24   5  10
           13  12  28  21  24   8
           14  25   8   7   2  27
           15   5  14   9   8   1
           16   4   6  12  25   3
           17   8  17  13  29  23
           18  22   9  17  26   4
           19  24  11  23  27  19
           20  17  20  27   3  14
           21  21  10  14  13  16
           22   9  30   2  19   6
           23  19  21  20   1  29
           24  30  25  16  23  22
```
