# Supplementary material for: Rectangular Augmented Row–Column Designs Generated From Contractions
Source: Biom J. 2026 Sep 24;68(5):e70182. doi: 10.1002/bimj.70182 (PMC13613013; doi:10.1002/bimj.70182)
Supplement: Supplementary file 2 [file BIMJ-68-e70182-s003.zip › README.pdf]

README instructions for the results in the paper

Rectangular augmented row-column designs generated from contractions

by Hans-Peter Piepho and Emlyn Williams

The efficiency factor calculations for the designs in Table 1 of this paper (which are listed in Supplementary B.docx) can be obtained using the Genstat (version 24) program Table 1_calculations.gen.
Spreadsheet versions of the designs (con(v,k,s),xlsx) are included in the zip file. For example the first design listed in Table 1 would correspond to the file con(12,3,8).xlsx.
In the Genstat program, open the desired spreadsheet and change the first line to match the appropriate values for v, k and s.
The values for columns 5-8 of Table 1 are returned by running the program.

Spreadsheet forms for Examples 1 and 2 (Supplementary B.xlsx) are also included in the zip file, namely con(12,3,8).xlsx and con(24,5,16).xlsx and efficiency factors can be obtained using the same Genstat program.

The program runs instantaneously.
