# Supplementary material for: Rectangular Augmented Row–Column Designs Generated From Contractions
Source: Biom J. 2026 Sep 24;68(5):e70182. doi: 10.1002/bimj.70182 (PMC13613013; doi:10.1002/bimj.70182)
Supplement: Supplementary file 2 [file BIMJ-68-e70182-s003.zip › con(12,3,8).rtf]

  32
  33  \ open the spreadsheet file con(v,k,b).xlsx and change the parameters in the next line to match the example
  34  \ for example the next line is currently set to read the input file con(20,5,16).xlsx
  35
  36  scalar [8] s & [12] v & [3] k
  37  calc b=s & n=b*k
  38  matrix [n;b] nb
  39  for i=1...n
  40  for j=1...b
  41  calc nb$[i;j]=1*(column$[i].eq.j)
  42  endfor
  43  endfor
  44  matrix [n;k] nk
  45  for i=1...n
  46  for j=1...k
  47  calc nk$[i;j]=1*(row$[i].eq.j)
  48  endfor
  49  endfor
  50  matrix [n;v] nv
  51  for i=1...n
  52  for j=1...v
  53  calc nv$[i;j]=1*(treatment$[i].eq.j)
  54  endfor
  55  endfor
  56  calc ki=ltproduct(nb;nb) & ri=ltproduct(nv;nv)& bi=ltproduct(nk;nk)
  57  calc nt1=ltproduct(nv;nb) & nt2=product(nt1;inverse(ki)) & nt3=rtproduct(nt2;nt1)
  58  calc nt11=ltproduct(nv;nk) & nt22=product(nt11;inverse(bi)) & nt33=rtproduct(nt22;nt11)
  59  vari [nv=n] one1
  60  calc one1=1
  61  calc r1=ltproduct(nv;one1)
  62  calc rr=rtproduct(r1;r1)
  63  calc a=ri-nt3-nt33+rr/n
  64  calc nt4=sqrt (inverse(ri))& nt5=product(nt4;a)
  65  calc astar=product(nt5;nt4)
  66  calc arcb=ri-rr/n
  67  calc ev1mod=evalues (a)
  68  \print ev1mod
  69  calc ev1=evalues(astar)
  70  \print ev1
  71  scalar rsum
  72  calc rsum=0
  73  for i=1...v
  74  calc rsum=rsum+ri$[i;i]
  75  endfor
  76  calc rav=rsum/v
  77  \print rav
  78  scalar ind1,vm1
  79  calc ind1=0 & vm1=v-1
  80  for i=1...vm1
  81  calc ind1=ind1+1/ev1mod$[i]
  82  endfor
  83  calc ef1=vm1/(ind1*rav)
  84  \print ind1;dec=4
  85  \print ef1;dec=4
  86
  87  calc mt1=ltproduct(nb;nv) & mt2=product(mt1;inverse(ri)) & mt3=rtproduct(mt2;mt1)
  88  calc mt11=ltproduct(nb;nk) & mt22=product(mt11;inverse(bi)) & mt33=rtproduct(mt22;mt11)
  89  calc k1=ltproduct(nb;one1)
  90  calc kk=rtproduct(k1;k1)
  91  calc ma=ki-mt3-mt33+kk/n
  92  calc mt4=sqrt (inverse(ki))& mt5=product(mt4;ma)
  93  calc mastar=product(mt5;mt4)
  94  calc mev1=evalues(mastar)
  95  \print mev1
  96
  97
  98  scalar mind1,mvm1
  99  calc mind1=0 & mvm1=b-1
 100  for i=1...mvm1
 101  calc mind1=mind1+1/mev1$[i]
 102  endfor
 103  calc mef1=mvm1/mind1
 104  \print mind1;dec=4
 105  \print mef1;dec=4
 106
 107  calc vstar=v*b-k*(b-1) & w=vstar-(v+b)+1
 108  calc etemp=(b-1)/mef1+(v-1)/ef1 & eaug=(vstar-1)/(w+etemp*v/k)
 109  \print eaug ; dec=4
 110  calc W=rtproduct(nt11;nt11)
 111  calc extra=ri-W/b & extra2=mt1-product(mat1(b;v);ri)/b
 112  calc mt2mod=product(extra2;inverse(extra+mat1(v;v)))& mt3mod=rtproduct(mt2mod;extra2)
 113  calc mastarmod=ident(b)-mt3mod/k
 114  calc adjust=product(mat1(b;b);mastarmod)/b
 115  calc mastarmodadj=mastarmod-adjust
 116  \print nt1;field=5;dec=2
 117  calc WN=product(W;nt1)
 118  \print W; field=4;dec=1
 119  \print WN;field=4;dec=1
 120  calc mev1mod=evalues(mastarmodadj)
 121  \print mev1mod
 122
 123  calc mind1mod=0
 124  for i=1...mvm1
 125  calc mind1mod=mind1mod+1/mev1mod$[i]
 126  endfor
 127  calc mef1mod=mvm1/mind1mod
 128  \print mind1mod;dec=4
 129  \print mef1mod;dec=4
 130
 131  calc indsum=ind1+mind1mod
 132  \print indsum;dec=4
 133
 134  calc vstar=v*b-k*(b-1) & w=vstar-(v+b)+1
 135  calc etemp=(b-1)/mef1mod+(v-1)/ef1 & eaugmod=(vstar-1)/(w+etemp*v/k)
 136  \print eaugmod ; dec=10
 137
 138  calc E_con=ef1 & Cbar_s=mef1mod & E_dual=mef1 & E_aug=eaugmod
 139  print E_con,Cbar_s,E_dual,E_aug;field=7;dec=4,4,4,6
 
	E_con	Cbar_s	E_dual	 E_aug
	0.5739	 0.4828	 0.4828	 0.388112
 
 140
